# Successful Outpatient Treatment of Legionella pneumophila Pneumonia With Lascufloxacin: A Case Report

**DOI:** 10.7759/cureus.81207

**Published:** 2025-03-25

**Authors:** Jun Hirai

**Affiliations:** 1 Division of Infection Control and Prevention, Nippon Medical School Chiba Hokusoh Hospital, Inzai, JPN

**Keywords:** a-drop, lascufloxacin, legionella pneumophila, outpatient, pneumonia

## Abstract

*Legionella pneumophila* is a significant cause of community-acquired pneumonia (CAP) and often necessitates hospitalization. Lascufloxacin (LSFX), a novel fluoroquinolone with high pulmonary penetration, has demonstrated efficacy in treating *Legionella* pneumonia in hospitalized patients. However, its use in an outpatient setting for *Legionella* pneumonia has not been previously documented, despite its favorable pharmacokinetics and safety profile. We present a 49-year-old man with a history of smoking and dyslipidemia who developed a high fever, headache, and malaise. In addition to the presence of relative bradycardia, chest imaging revealed right lower lobe pneumonia, and a urinary antigen test confirmed *Legionella pneumophila* infection. Despite this diagnosis, his condition remained stable (A-DROP score: 1), allowing for outpatient management. He was prescribed LSFX 75 mg/day for 10 days, with close monitoring via home pulse oximetry and scheduled frequent follow-up visits. His fever was resolved by day 3, and he fully recovered without complications or adverse effects. This is the first reported case of successful outpatient treatment of *Legionella* pneumonia with LSFX. The decision for outpatient therapy was based on the patient’s stable condition based on A-DROP (age, dehydration, respiratory failure, orientation disturbance, and low blood pressure) scoring, LSFX’s excellent bioavailability and pulmonary penetration, and its lack of renal dose adjustment requirements. However, the A-DROP scoring system may underestimate *Legionella* pneumonia severity, necessitating careful patient selection. LSFX appears to be a safe and effective option for outpatient management of mild *Legionella* pneumonia. This case highlights its potential as an alternative to inpatient treatment, but further studies are required to confirm its broader applicability.

## Introduction

*Legionella pneumophila* is a Gram-negative, facultative intracellular bacterium and the primary cause of Legionnaires' disease, a severe form of community-acquired pneumonia (CAP) associated with significant morbidity and a mortality rate of approximately 10-30% without prompt appropriate therapy [1]. *Legionella* is typically acquired through inhalation of contaminated water sources such as hot springs, cooling towers, and water distribution systems [2]. Due to its intracellular nature, effective treatment requires antibiotics with strong intracellular penetration, such as fluoroquinolones (FQs) and macrolides (MCs) [3].

Lascufloxacin (LSFX), a novel 8-methoxy fluoroquinolone with a broad spectrum of antibacterial activity, is a new quinolone antibiotic that was approved in Japan in May 2020 and launched in June for the treatment of respiratory tract infections, including CAP and aspiration pneumonia [4]. Compared to older FQs like levofloxacin (LVFX), LSFX exhibits superior lung tissue penetration, achieving high concentrations in epithelial lining fluid and alveolar macrophages, critical sites for *Legionella* infection [5]. Additionally, LSFX has demonstrated favorable pharmacokinetic and safety profiles, with a lower risk of central nervous system toxicity and QT prolongation compared to other FQs [6,7]. Of these characteristics, LSFX, along with LVFX, is listed as a first-line antibiotic choice for *Legionella* pneumonia in the Japanese Respiratory Society (JRS) Guideline for the Management of Pneumonia in Adults, 2024 [8] and the Japanese Academy for International Dentistry/Japanese Society of Chemotherapy Guide to Clinical Management of Infectious Diseases, 2023 [9].

Outpatient management of pneumonia offers several advantages, including reduced healthcare costs, a lower risk of nosocomial infections, and improved patient satisfaction [10]. However, selecting patients for outpatient treatment requires careful assessment of disease severity and risk factors to ensure safe and effective therapy.

While previous studies have reported successful LSFX treatment for *Legionella* pneumonia in hospitalized patients [11], its use in an outpatient setting has not yet been documented. This case report presents the first successful outpatient treatment of *Legionella pneumophila* pneumonia with LSFX in an adult patient with mild disease severity. To the best of our knowledge, this is one of the first reports demonstrating LSFX as an effective outpatient therapy for Legionnaires' disease, highlighting its potential as a safe and practical alternative to conventional treatment regimens.

## Case presentation

This case involves a 49-year-old man with dyslipidemia and a history of smoking for over 30 years. He presented to the hospital with a high fever exceeding 40°C, worsening headache, and progressively reduced food intake. He had been experiencing fever, headache, and joint pain for one week. On arrival, his level of consciousness was normal and his vital signs were as follows: body temperature, 40.4°C; blood pressure, 142/70 mmHg; pulse, 80 beats/min; respiratory rate, 12 breaths/min; and oxygen saturation (SpO_2_), 96% on ambient air. Physical examination revealed no conjunctival congestion, cervical lymphadenopathy, neck stiffness, or positive jolt sign. There was no abdominal pain. On chest auscultation, no heart murmurs were noted, but late crackles were heard in the right posterior lung field.

A nasopharyngeal swab test for influenza antigens (ImunoAce Flu, TAUNS, Shizuoka, Japan) was negative, as was the SARS-CoV-2 antigen test (ImunoAce SARS-CoV-2, TAUNS, Shizuoka, Japan). Blood tests showed an elevated white blood cell count (13,400/µL; neutrophils, 85.5%), elevated C-reactive protein (21.5 mg/dL), elevated aspartate aminotransferase (AST, 56 U/L), alanine aminotransferase (ALT, 47 U/L), and lactate dehydrogenase (LDH, 388 U/L), as well as hyponatremia (132 mmol/L). Blood urea nitrogen was elevated (25 mg/dL), while serum creatinine was slightly elevated at 1.0 mg/dL. Serum creatine kinase levels were normal.

**Figure 1:**
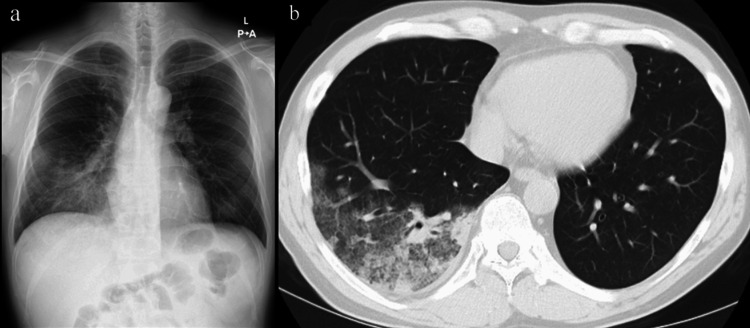
Chest x-ray and chest CT of the present case on admission to our hospital. (a) The frontal chest radiograph shows a patchy consolidation in the lower lobe of the right lung; (b) the axial chest CT (lung window) shows a multifocal consolidation with air bronchograms in the right lower lobe, accompanied by ground-glass opacities.

A chest x-ray revealed an infiltrative opacity in the right lower lung, and a chest computed tomography (CT) scan confirmed lobar pneumonia (Figure 1).

Given the presence of relative bradycardia (a heart rate lower than expected for the level of fever) and the chest imaging findings, *Legionella* pneumonia was suspected. Further investigation revealed that the patient had traveled to a hot spring six days before the onset of symptoms. In addition, *Legionella pneumophila *pneumonia was strongly suspected based on the *Legionella *pneumophila prediction score established in Japan [12] (Table 1; a total score ≥3 has a sensitivity of 93% and a specificity of 75% for *Legionella* pneumonia), *Legionella* pneumonia was strongly suspected. The diagnosis was confirmed by a positive *Legionella *urinary antigen test using BinaxNOW Legionella (Abbott Diagnostics Medical, Lake Forest, CA, USA). The patient's general condition was relatively stable, with an A-DROP (age, dehydration, respiratory failure, orientation disturbance, and low blood pressure) score of 1, which indicates a mild severity of illness for this patient (Table 1).

**Table 1:** Legionella pneumophila pneumonia prediction score and A-DROP score of the present case. A-DROP: Age, dehydration, respiratory failure, orientation disturbance, and low blood pressure; SpO_2_: Oxygen saturation.

	This case	Score
Legionella pneumophila pneumonia prediction score		
Male	Present	1
Without cough	Present	1
Dyspnea	Absent	0
C-reactive protein≥18 mg/dL	Present	1
Serum sodium concentration<134 mmol/L	Present	1
Serum lactate dehydrogenase≥260 U/L	Present	1
Total score	-	5
A-DROP score		
Age≥70	Absent	0
Blood urea nitrogen≥21 mg/dL	Present	1
SpO_2_≤90%	Absent	0
Consciousness disorder	Absent	0
Systolic blood pressure≤90 mmHg	Absent	0
Total score	-	1

A-DROP is a severity scoring system used in Japan for assessing CAP, developed by the JRS [8]. It is an adaptation of the CURB-65 score, which is commonly used worldwide, but A-DROP is modified to better fit the Japanese population, particularly elderly patients [8]. Given his desire to return home and the availability of a pulse oximeter for SpO_2_ monitoring in his home, we instructed him to return to the hospital immediately if a significant SpO_2_ decrease occurred (e.g., a sustained level below 94%). After obtaining two sets of blood cultures and prescribing LSFX, he returned home.

No sputum culture or polymerase chain reaction (PCR) testing was performed because the patient did not produce sputum despite repeated attempts. Although molecular or culture-based confirmation would have strengthened microbiological validation, the diagnosis of *Legionella pneumophila* pneumonia was based on a positive urinary antigen test and supported by clinical findings such as hyponatremia, elevated LDH, and compatible chest imaging, fulfilling widely accepted diagnostic criteria. Outpatient follow-up visits on days 2, 7, and 10 confirmed that he experienced no respiratory distress or decline in SpO_2_. LSFX was administered at 75 mg once daily for a total of 10 days. His fever was resolved by the third day of LSFX treatment, though he developed a transient dry cough, which resolved by day 10. The seven-day blood culture was negative. By the 10th day of treatment, his white blood cell count, sodium levels, AST, ALT, and LDH had normalized. No side effects related to LSFX, such as gastrointestinal symptoms, were observed.

## Discussion

To date, there have been no reports of the outpatient use of LSFX in cases of *Legionella* pneumonia, and this report provides valuable data for evaluating its efficacy and safety. The patient had typical risk factors for *Legionella *pneumonia such as a recent hot spring trip, and typical vital signs (relative bradycardia), blood test (hyponatremia), and lobar pneumonia, leading to early suspicion of *Legionella *pneumonia. The diagnosis was confirmed by a positive urinary antigen test, allowing for targeted antimicrobial therapy by LSFX.

The decision to treat the patient with LSFX as an outpatient was based on multiple factors. First, the patient had a mild disease severity by A-DROP score and had a pulse oximeter for SpO_2_ monitoring in his home in addition to several follow-up hospital visits. Second, no dose adjustment is required for LSFX in patients with renal impairment (the present case had a slightly higher creatinine level). Third, LSFX has demonstrated excellent bioavailability, pulmonary penetration, and intracellular accumulation in alveolar macrophages, making it an ideal candidate for treating *Legionella* pneumonia. For instance, with a single dose of LVFX 500 mg, the concentration in the alveoli is approximately five times the concentration in the blood [13], but with a single dose of LSFX 75 mg, the concentration in the alveoli is approximately 38 times the concentration in the blood [14].

In this case, LSFX was used for successful outpatient treatment; however, whether this choice was optimal is debatable. First, there is still no clear consensus on whether FQs or MCs should be chosen for the treatment of *Legionella* pneumophila. However, recent studies have compared the therapeutic efficacy of these antibiotics and found a meta-analysis reported that FQs significantly reduced 30-day mortality compared to MCs (odds ratio 0.41, 95% confidence interval 0.20-0.85) [15]. In addition, it has been reported that the fluoroquinolone treatment group shows faster defervescence and clinical stabilization than the macrolide treatment group (e.g., defervescence and stabilization in the LVFX group in an average of two to three days and in the macrolide group in four to five days) [16]. Second, there is the clinical question of whether choosing LSFX, which has less clinical data, over CPFX or LVFX was a prudent decision. Although no clear evidence demonstrates the superiority of one fluoroquinolone over another, previous Japanese clinical trials have found no significant differences in the efficacy of CPFX, LVFX, garenoxacin, and moxifloxacin, all of which are oral drugs [17]. These studies reported high cure rates and favorable safety profiles for all these FQs in treating *Legionella* pneumonia [17]. International guidelines also recommend any of these FQs as first-line treatment for *Legionella* pneumonia [3]. In regard to adverse events, in a clinical trial conducted in Japan [18], the incidence of drug-related adverse events was approximately 11.7% among 531 patients who received 75 mg of LSFX. In comparative studies, the incidence of adverse events was 11.8% in the LSFX group and 14.5% in the LVFX group, suggesting a lower incidence with LSFX. Notably, fluoroquinolone-associated adverse events, such as gastrointestinal and central nervous system symptoms, occurred less than half as frequently with LSFX as with LVFX (e.g., gastrointestinal disorders: LSFX 3.2% vs. LVFX 7.2%; central nervous system disorders: LSFX 0.4% vs. LVFX 1.8%) [18]. No serious adverse events were observed during the study, and no fluoroquinolone-specific adverse effects, such as photosensitivity, QT prolongation, abnormal blood glucose levels, or tendon disorders, were reported [18]. Based on these findings, LSFX is considered safe for outpatient use and carries a lower risk of adverse events compared to traditional FQs. Furthermore, as an oral agent, LSFX could be an effective treatment option for mild cases of *Legionella* pneumonia, such as in this case.

Limitations

Although the patient tolerated LSFX well with no significant side effects, this case report has several limitations. First, the A-DROP scoring system, which was used to assess disease severity in this case, has been reported to underestimate the severity of *Legionella* pneumonia compared to other scoring systems, such as the Infection Disease Society of America (IDSA) guidelines [19]. In a previous study, 10 of 15 cases were classified as intermediate, three as severe, and two as extremely severe using the A-DROP system, whereas most cases were classified as severe according to the IDSA guidelines. Among five fatal cases, three were ranked as intermediate using the A-DROP system, whereas all fatal cases were classified as severe by the IDSA guidelines [19]. This limitation should be considered when evaluating the appropriateness of outpatient management for *Legionella* pneumonia based on A-DROP. Given its lower sensitivity in assessing severity compared to other scoring systems, such as the American Thoracic Society (ATS)/IDSA guidelines, reliance solely on A-DROP may lead to under-triage of patients who might otherwise benefit from inpatient monitoring [20]. Second, in this case, the patient was treated with LSFX for a total of 10 days, during which time clinical stability was confirmed. However, the optimal duration of treatment remains unclear, and further studies are needed to establish standardized treatment protocols. Third, the diagnosis of *Legionella pneumophila* pneumonia was based solely on a positive urinary antigen test without culture or PCR confirmation. However, the patient had a typical clinical history, and the vital signs and blood test results strongly supported the diagnosis of *Legionella* pneumonia. Finally, as this is a single case report, generalization of the findings should be approached with caution. Our primary aim is not to draw broad conclusions but to present a unique and clinically relevant instance of the successful outpatient use of LSFX for *Legionella* pneumonia. Larger studies are necessary to evaluate the efficacy and safety of LSFX in broader outpatient populations.

## Conclusions

This case suggests the first documented outpatient treatment of *Legionella pneumophila* pneumonia with LSFX, demonstrating its potential as a safe and effective therapeutic option. The patient's mild disease severity, adequate home monitoring with a pulse oximeter, and close outpatient follow-up contributed to the successful outcome. While limited by its nature as a single case, it suggests that LSFX may serve as a safe and effective therapeutic option in carefully selected patients. Further studies are needed to validate these findings.
